# Negotiating the Unknown: Lessons Learned From Australian Healthcare Professionals Working Through the COVID19 Pandemic

**DOI:** 10.1155/jonm/2722679

**Published:** 2026-04-03

**Authors:** Amy-Louise Byrne, Robyn Preston, Pauline Calleja, Dawn Cameron, Stacey George, Rebecca Munt, Barbra Zupan, Shannon Delport, Samantha Jakimowicz, Clare Harvey, Janie Brown

**Affiliations:** ^1^ The International Consortium on Occupational Resilience, School of Nursing, Paramedicine and Healthcare Sciences, Charles Sturt University, Bathurst, New South Wales, Australia, csu.edu.au; ^2^ School of Nursing, Midwifery and Social Science, CQUniversity, Sydney, New South Wales, Australia, cqu.edu.au; ^3^ School of Health and Applied Science, CQUniversity, Townsville, Queensland, Australia, health.qld.gov.au; ^4^ School of Nursing, Midwifery and Social Science, CQUniversity, Townsville, Queensland, Australia, health.qld.gov.au; ^5^ College of Healthcare Sciences, James Cook University, Townsville, Queensland, Australia, health.qld.gov.au; ^6^ School of Health and Life Sciences, University of West Scotland, Glasgow, UK, uws.ac.uk; ^7^ College of Nursing and Health Sciences, Flinders University, Adelaide, South Australia, Australia, flinders.edu.au; ^8^ Adelaide Nursing School, The University of Adelaide, Adelaide, South Australia, Australia, adelaide.edu.au; ^9^ School of Graduate Research, CQUniversity, Rockhampton, Queensland, Australia, health.qld.gov.au; ^10^ School of Health Medical and Applied Sciences, CQUniversity, Rockhampton, Queensland, Australia, health.qld.gov.au; ^11^ School of Nursing, Paramedicine and Healthcare Sciences, Charles Sturt University, Bathurst, New South Wales, Australia, csu.edu.au; ^12^ School of Nursing and Midwifery, Massey University, Albany, New Zealand, massey.ac.nz; ^13^ School of Nursing, University of British Columbia, Okanagan, British Columbia, Canada, ubc.ca; ^14^ Curtin School of Nursing, Curtin University, Perth, Western Australia, Australia, curtin.edu.au; ^15^ St John of God Midland Public and Private Hospital, Midland, Western Australia, Australia; ^16^ The Western Australian Group for Evidence Informed Health Practice: A JBI Center of Excellence, Curtain University, Perth, Western Australia, Australia

**Keywords:** COVID-19, disaster, nursing, organizational responses, well-being

## Abstract

**Aim:**

To explore the experiences of healthcare professionals who worked in patient‐facing roles during the pandemic.

**Design and methods:**

This study, conceptualized at the beginning of the pandemic response in Australia, aimed to understand healthcare professionals’ experiences regarding the professional and personal impacts of working through the COVID‐19 pandemic. Nine healthcare professionals participated in semistructured interviews. Thematic analysis resulted in four themes. The COREQ checklist guided the process (see Supporting File 1).

**Results:**

The overall themes included (1) Little Whispers, Moving into A Pandemic; (2) Confusion and Chaos; (3) Pushing Boundaries and Finding Ways of Coping; and (4) Stories of Trauma, Stories of Opportunity. Findings suggest that healthcare professionals were initially naïve about the significance of the pandemic and experienced increased feelings of vulnerability as its impact became clearer. A lack of consistent messaging was reported to increase feelings of confusion and chaos.

**Conclusion:**

To cope, healthcare professionals reported relying on each other and worked to identify innovative solutions to care. Stories highlight complex responses to the increased pressures and expectations of working through the pandemic, opening a space for future research.

**Contribution to nursing:**

This study provides insight into the experiences of Australian healthcare professionals working directly with patients during the COVID‐19 pandemic. The findings suggest that participants experienced feelings of vulnerability and fear as they grew to understand the risks of the pandemic. The findings from this study can contribute to lessons learned from the pandemic to enable preparedness for future pandemic responses.

**Reporting method:**

COREQ.

## 1. Introduction

The COVID‐19 pandemic differentially affected countries across the world, with a wide range of government responses and restrictions enforced in community and hospital settings. Governments responded swiftly, mandating restrictions and behaviors in communities and implementing additional infection control measures in healthcare settings [1]. Australia was able to learn from what was unfolding in other countries and take a preventative response in its recommended measures [2]. Nonetheless, there were mandates and advice coming from both state and federal health departments. In addition to being fragmented [3], the mandates and advice resulted in rapid change for healthcare professionals (HCPs), significantly altering the conditions in which they work and their approach to care [4].

The pressure of rapid change was felt across healthcare systems, resulting in high job demands, tumultuous working conditions, and increased psychological stress and anxiety, which increased turnover intention, exacerbating shortage and retention issues [5]. Studies conducted early in the pandemic indicated that HCPs were at an increased risk of negative psychological consequences. In a large‐scale survey (*n* = 9518 participants), more than 50% of respondents reported symptoms of anxiety and depression [6]. Symptoms of posttraumatic stress disorder (PTSD) have also been reported [7].

Healthcare systems across the country were significantly challenged by the pressures related to preparing for the expected and unexpected effects of the continuing pandemic [8], and nurses, who work at the forefront of the healthcare system, were reported to be experiencing higher levels of anxiety than prevalence ratings of nurses globally [9]. Fernandez et al. [9] suggested that the increased prevalence of anxiety among nurses in Australia may be due to exposure to ever‐worsening conditions elsewhere and the increased time to ruminate about anticipated risks. However, contributing factors were not examined; thus, the factors impacting the well‐being of HCPs in Australia required further exploration.

Retaining HCPs is essential in such uncertain times to ensure the efficient, safe care of those requiring it. Further workforce pressure, especially those experienced with the nursing shortage, places an increasing burden on health services.

Like nurses, allied health professionals are also at risk for occupational stress and potential burnout due to proximity to patients who require ongoing care, changing clinical practice guidelines, incorporation of new technologies such as telehealth into patient care, broadening of the scope of practice, and/or the requirement to change roles altogether to assist on the front lines. Thus, support for their resilience and well‐being is also necessary to reduce the attrition of these professionals in the workforce.

HCPs were at the forefront of the COVID‐19 response, working under immense pressure and uncertainty. Their experiences offer critical insights into how healthcare systems function during crises, how policies are enacted on the ground, and how individuals and teams adapt in real time. Listening to frontline workers is essential not only to understand the human impact of the pandemic but also to inform the adaptation of healthcare structures and systems [10]. Their reflections can guide the development of more responsive, resilient models of care and ensure that support mechanisms are in place to protect their well‐being in future unprecedented events. This study centers on those voices.

This research was conceptualized at the beginning of the pandemic response, as a contemporary understanding of the psychological impact of working through a pandemic was unknown. It was anticipated that negotiating the pandemic could transform into psychological harm to HCP. While research on this topic was quick to surface, most explored the impacts on HCP well‐being without the benefit of hindsight, and many were survey‐based. The specific aim of this study was to explore the experiences of HCPs working through the COVID‐19 pandemic, to better understand professional and personal factors impacting their well‐being, conducted post pandemic.

## 2. Methodology

This article presents the qualitative findings (phase two) of a larger study, which aimed to explore the impact of the worldwide COVID‐19 pandemic on clinically based nurses’, midwives, allied health professionals, and students of these disciplines professional and personal well‐being. The research utilized a prospective, longitudinal design to collect data (quantitative and qualitative) across three time points (start of pandemic, peak, and end of pandemic) via survey. Surveys were disseminated via social media and professional networks. Within these surveys, participants could provide an email address to be contacted for phase two of the study, where they engaged in a qualitative one‐on‐one interview.

This phase of the study aimed to explore the impact of the COVID‐19 pandemic on the professional and personal well‐being of Australian nurses, midwives, allied health professionals, and students of these disciplines.

The objective was to:

1. Capture the experiences of health professionals who worked in patient‐facing roles through the COVID‐19 crisis (postpandemic).

This study employed a qualitative design using semistructured interviews, as described by DeJonckheere and Vaughn [11] to explore the experiences of HCPs during the COVID‐19 pandemic. Interviews were conducted postpandemic to allow for reflection with the benefit of hindsight. The use of COREQ [12] checklist supported transparency and completeness in reporting.

### 2.1. Ethical Considerations

Ethical approval for this research was obtained through Curtin University Human Research Ethics Committee (HREC), number EC00262, with reciprocal approval achieved via the HRECs at each university.

### 2.2. Recruitment and Sampling

Phase two of the study (the focus of this article) consisted of one‐on‐one interviews exploring the stories and experiences of HCP working during the pandemic. A total of 27 individuals indicated interest in participating via the survey. All were provided with a copy of the information sheet and consent form via email. Eleven people consented; however, two people did not attend the interview resulting in a total of nine (*n* = 9) interviews. All participants identified as female. Most participants were senior registered nurses, and one clinical pharmacist participated. All participants were patient facing. While the sample size of nine (9) participants is relatively small compared to the broader population of HCPs affected by the pandemic, each participant offered deeply valuable insights. Their willingness to share personal and professional experiences provides rich, meaningful data that undoubtedly contributes to our understanding of the healthcare workforce’s challenges during COVID‐19. Indeed, Hennick and Kaiser [13] suggested that qualitative sample sizes of between 9 and 17 participants is often enough to achieve meaningful insights.

### 2.3. Data Collection and Analysis

One‐on‐one postpandemic semistructured interviews were conducted between May 18 and July 14, 2023 via the Teams videoconferencing platform and were recorded and transcribed in real time. All interviews were conducted by the lead author (female), who is a senior lecturer and experienced at qualitative interviews; only this researcher and the participant was present at each interview. The researcher maintained awareness of her professional background and potential biases throughout the interviews. No existing relationships between the researcher and participants existed, and participants were informed of the study aims and objectives prior to the interview via the information sheet. Given the busy lives of HCPs, interviews were between 28 and 39 min in duration. Guiding questions were used as prompts; however, participants were encouraged to discuss their experiences freely.

A thematic analysis was conducted. Braun and Clarke’s [14] six‐step process was followed, which included (a) familiarization with the data, (b) coding, (c) searching for themes, (d) reviewing themes, (e) defining and naming themes, and (f) writing‐up. Initial inductive analysis was independently conducted by AB, RP, and DC on a purpose‐built Excel spreadsheet. The spreadsheet was tested with three interviews, and minor adjustments were made prior to completion of initial analysis. Consensus was reached on the themes via a team meeting, and themes were consolidated and collapsed. The final stages of analysis included the consideration and naming of final themes, with consistency noted between data and findings. Data validation against the themes was conducted by SG.

To maintain rigor, the study employed several strategies, including detailed documentation of coding decisions and peer review of codes, categories, and themes within the research team. Reflexivity was maintained through an ongoing discussion of researcher positionality and potential biases.

Analysis identified four key themes: (1) Little Whispers, moving into a Pandemic; (2) Confusion and Chaos; (3) Pushing Boundaries and Findings Ways of Coping; and (4) Stories of trauma/Stories of Opportunity.

## 3. Findings

An overview of the participants is provided in Table 1.

**TABLE 1:** Research participants.

Pseudonym	Profession	Clinical area	Public or private	Australian state
Bobbi	Registered Nurse	Mental health Community	Private	WA
Kerri	Registered Nurse	Emergency	Public	NSW
Fiona	Registered Nurse	Neonatal Intensive Care Unit	Private	WA
Maeve	Clinical Pharmacist	Community	Public	SA
Tracy	Registered Nurse	Older persons mental health	Public	SA
Louise	Registered Nurse	Emergency	Public	NSW
Helen	Registered Nurse	Respiratory Community and outpatient	Public	NSW
Margaret	Registered Nurse	Breast Cancer nurse community	Private	WA
Adele	Registered Nurse	Rural Hospital	Public	WA

**FIGURE 1:**
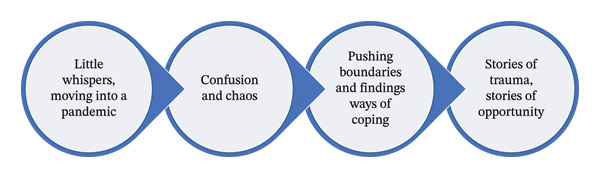
Health professional experiences of working during the COVID‐19 pandemic.

To best capture the stories and nuances of the participants involved in this study, the findings are presented in narrative form and summarized in Figure 1.

### 3.1. Little Whispers, Moving Into a Pandemic

Participants recalled first hearing about the pandemic as a seminal moment for them. Most discussed learning about it through media and news outlets, and the virus becoming more prevalent in various forms of discourse. While all participants talked about hearing of the virus, they collectively reported not understanding the significance it would have, and the rapid changes that would occur to the system, their professional and personal lives.

Indeed, in the early days and initial spread of the virus, one participant described the information as “little whispers” indicating that the impact was not yet understood:

*I bought my house in the January of 2020, you know, and COVID wasn’t really, like there was little whispers. …that was about China or Wuhan or something—*Bobbi

While news of the virus became more widely reported, participants stated that they initially lived life as normal. Some participants talked about buying a home, planning holidays, and planning to move locations, not foreseeing those measures such as lockdowns, stay at home orders, and zoning (where people were asked to stay within a certain radius of their homes) would be mandated. All participants talked about hindsight, and how they might have been better prepared if they had known the measures that would be put into place, for example:

*I wasn′t ready to move for six or eight months. It’s a big move and so I rented the house out and stayed in Melbourne, not knowing, you know, hindsight would have been a wonderful thing—*Kerri

As the reality of the pandemic became clearer, with increasing knowledge of international death rates, hospitals beginning to be overrun, and HCP becoming ill, our participants reported that they started to feel vulnerable and at times fearful for themselves, their family and community. Participants described this as a stressful time, with many unknowns. Factors such as age, family living in dispersed locations, and being overseas were prominent concerns raised by participants:

*…and because I was older…I was 60 and I was feeling quite vulnerable—*Bobbi

Our participants did not speak of any particular work‐based changes in the early stages of the virus but described work as business as usual. However, in March 2020, the World Health Organization declared COVID‐19 a global pandemic, and Australia declared a national lockdown [15]. All nonessential services were temporarily ceased, border controls were put in place, and citizens were directed to stay home. These actions led to a period of confusion and chaos, the second theme, as described by the participants.

### 3.2. Confusion and Chaos

Once the pandemic was declared, and state and federal governments began putting measures in place, participants described a difficult period of confusion and chaos. In the context of strict lockdowns and rapidly changing health directives, participants described this as a period of intense turmoil and change.

All participants reported a lack of, or fractured, flow of information as a source of anxiety and stress in the workplace. Confusion and rapid change were common themes, and participants in this study reported being unsure of what were correct protocols. Those in community roles reported that information was hospital centric, leaving community‐based providers feeling out of the loop and isolated. Some health services temporarily ceased community‐based care; however, some did continue, for example, due to the medications that their patients required.

Participants described a distinct lack of information flow, as described by one individual:

*At the time, I was full time nights and the information we heard was like Chinese whispers, but [I had to] look it up for myself, to find out exactly what the story was—*Margaret

This lack of information and its confusing messages had a flow on effect to clinical care. Participants described an additional burden on clinical care, where personal protective equipment requirements meant that providing care and antibiotics took considerable time. Not only was this a requirement in hospitals but also in people’s homes. Many felt this created fear, distrust for patients, and neighbors. In the hospital context, one participant talked of a cardiac arrest where HCPs were reluctant to intervene due to COVID‐19 protocols:

*I watched people stand around with a woman who was arresting and because the protocol had been that any intubation required [that] you get the anaesthetic team down in their full PPE. They delayed doing her airway for a good while and I was like, I′m in full PPE. I′ve been bagging patients my all my life and I picked up a bag—*Kerri

One participant indicated that clerical staff were directed away from direct patient contact, and that this directive added extra responsibilities to (dominantly) nursing teams. Lack of consistent information was described as complex by the participants, as information streams came from government, health services, local hospital wards, and leaders. Participants expressed inconsistencies in the application of information between government to health services and health service to departments:

You know, they’d send memos around and about updates or around, you know, what the government was saying. And that? But then they wouldn’t follow what the government was recommending anyway—Bobbi.

Patients were also being provided information through public avenues such as the news, at times painting a picture that the hospital system was coping. The participants in this study felt that at times this information was deceptive and inaccurate, as described by the below participants:

*Information about how health care is being delivered. To not be filtered through. The gag orders, you know—*Kerri

*And*

*I suppose we were aware that the publicity around health care was propaganda pretty much saying we′re fine when we were already, we were already broken—*Louise

In addition to the communication issues, lack of, or scarcity of, resources added to the confusion and chaos. Participants talked about stockpiling PPE and having other departments take their supplies from them, leading to departments hiding and strictly controlling resources. One participant reported that toilet paper in public toilets at the hospital was being strictly controlled. They talked of running out of COVID‐19 tests and treatments, as described by the below participant:

*We almost ran out of COVID treatment a couple of times, but that′s from a Commonwealth [federal] level, so there was limited [stock] in the country—*Maeve

Staffing levels were described by participants as a constant issue. At times, wards were being run four to six nurses short. Some participants reported that HCP would simply not return, leaving their profession altogether. Senior staff/nurses felt compelled to nurture the staff/nurses that stayed, putting additional strain and responsibility on an already stretched workforce. One participant stated that staffing levels were already an issue before the pandemic, and COVID‐19 exacerbated this:

*The thing is, it hasn′t stopped because there′s still no staff and there′s, like, we had to train 16 new grads because they think that′s fine to put 16 new grads into ED the beginning of this year—*Tracey

In addition to limited physical resources, the lack of technology and innovative models of care were raised by participants. They reported that the health service was not prepared for the bulk of telehealth appointments and that many vulnerable people did not have the capability to utilize this form of care. Many patients missed appointments and opportunities to engage with their family:

*They don’t have the technology to keep in touch with family. No one was prepared in that respect—*Fiona

What was clear from the participants was that leadership within their respective areas was important to them and to the delivery of care. Participants talked about ineffective leadership and the need for upper management to be more visible during these difficult times. Some participants felt that management placed a higher value on budget over care at a time when HCPs felt compassion was most important:

*But there were things that I was finding out second or third hand that should have been coming from my managers, and information they′ve been sitting on, and they just hadn′t made a decision and you don′t have the luxury of sitting and making a decision in some of these things*—Maeve

*And*

*…but we really didn′t have much support and our manager…it was her first role as a team leader, and she wasn′t really good at it*—Bobbi

As a result of the fractures in the leadership, the study participants noted that staff/HCPs burned out, losing faith and confidence in themselves and the system:

*I watched medical seniors look so close to tears…I′ve never seen some of those self-opinionated, self-righteous people look so broken…the ones who look so cocky, they stop looking cocky…they just were struggling*—Kerri

### 3.3. Pushing Boundaries and Finding Ways of Coping

From the confusion and chaos came opportunities for HCPs to push existing boundaries and find new ways of working and coping. This included new care processes, changes to team dynamics and support structures, and finding workarounds to improve care.

The participants discussed the ways that they as individuals and as a collective work area innovated to create new care delivery processes. Some examples included improving telehealth outreach and COVID‐19 wards or areas in the emergency department. Two participants talked of particularly innovative solutions, such as using empty hospital beds for parents of children in the neonatal intensive care unit, as described below:

*Why can we not use the empty beds for the parents and keep them here so that at least they’re free to come and go see their baby? We know they’re safe. We did that for one of the lockdowns and that worked really well—*Fiona

In addition to this, one participant reported being enabled to set up a completely new service in the community for the assessment and treatment of antiviral medications in COVID‐19 patients:

*I had to set up whole new service and you know become an expert on all the antivirals and the treatments for mild COVID disease and then set up different pathways—*Maeve

At the same time, the restrictions implemented across society and health services increased referrals in the mental health sphere, especially for those in aged care. This was particularly difficult to manage due to lockdowns; however, one participant was proud to report that every person referred was called. Likewise, one participant talked about working around the structures for her community:

*I′d have friends who were really sick, and so I just wore my PPE from work, went into their homes, had to listen to their chests, got them the right kind of steroid… just so they could keep going*—Helen

Similarly, the participants talked about stepping up and being a source of support for their colleagues. Support was described in many forms, from being a shoulder to cry on, to backfilling leadership roles, staying for overtime or until every patient was seen, and offering peer support to junior HCPs, as described by the participants below:

*You spend a fair amount of time trying to nurture the ones that stayed—Kerri*

And

*if you′re a senior…you kind of shouldering the junior doctors because… the training of ED doctors is ridiculous—*Fiona

What naturally unfolded from the interviews were stories of trauma and stories of opportunities borne of the pandemic.

### 3.4. Stories of Trauma, Stories of Opportunities

Participants recounted their personal and professional stories of working during the COVID‐19 pandemic. One common theme was that of trauma and being traumatized by working during such a tumultuous time. The stories talk of stress, burnout, working extreme hours, and feeling obligated to help the health service. Many participants stated that the healthcare system was already fractured or broken and that the COVID‐19 pandemic exacerbated an already struggling system. Participants provided specific examples of what they went through, which included being performance managed, having a stroke, and leaving their long‐term job:

*It was actually quite traumatic as a nurse—*Fiona

*And*

*I′ve been [away from] there for a few months now, but I was quite burnt out and traumatised by the whole thing—*Adele

Participants talked about feeling abused by the service and patients, with little to no support available to them outside of generic employee assistance services, and that this led to many HCPs leaving:

*…[they say]…I′m not being abused for this, so I′m going to leave… It′s always frustrating when you′re losing staff because they′re hard to train up—*Fiona

The pressure and expectations on HCPs were also discussed at length, including the expectation that HCPs would return to work with COVID‐19 in full PPE and were expected to sign documents agreeing that they would not take their masks off. Not all HCPs agreed with such decisions and refused these processes, leading to further disruption and isolation. One participant described the health environment as “dangerous”:

*I’ve never noticed it as bad as I have over the last three years… to the point where nurses are leaving, like they’re saying that they’re stressed and leaving mid shifts—*Adele

Parallel to this, stories of opportunities were also present in the data. Some participants talked of being more physically active, as an outlet for stress, starting new social groups and finding new ways to connect. Examples included online bands, getting a dog, and seeking counseling. Other participants expressed that work became an escape from isolation where they could interact with people. Some participants commented that the pandemic provided an opportunity to learn how the system copes and what is missing, allowing for new infrastructure and models of care:

We’ve learned a lot about, like how our system cope with it means that our system is not *set* up for anything like this—Louise

*And*

*…there were these opportunities that opened up that were never there before. You weren′t able to do virtual online learning [prior]—*Bobbi

These findings demonstrate a complex and individualized journey of working through the COVID‐19 pandemic for those participating in the study. From feeling unprepared, thrust into confusion, and chaos, participants not only reported finding ways of coping but also reported widespread disruption and burnout. Despite this, some optimism remains, with the hope that the lessons learned from the COVID‐19 pandemic may advance healthcare delivery into the future.

## 4. Discussion

This study explored the stories of HCPs working during the COVID‐19 pandemic to describe how this impacted upon their health and well‐being. Participants described inadequacies in the Australian healthcare system and its ability to cope and respond to such a rapid change. They reported burnout, hospital services not coping with the rapid changes needing to be implemented, scarce physical resources, reducing HCP workforce, the number of changes being released within the healthcare system, and inaccurate information being publicized. These elements led to HCPs having to support each other and either innovate or leave the profession. Their stories and experiences offer important insights into how health services and leaders can better support staff through challenging and unexpected events.

The well‐being of frontline HCPs is not only a moral imperative but also a foundational requirement for a functioning health system. This study, alongside findings from Zhang and Gu [16], highlights that while some support mechanisms were introduced during the pandemic, many were short‐term and insufficient to address the enduring psychological toll. Participants in our study described burnout, trauma, and a sense of abandonment, with several recounting decisions to leave their roles or the profession entirely. This aligns with broader trends reported by Falatah [17] and Poon et al. [5], who documented increased turnover, and intentional and actual attrition among healthcare workers globally. The departure of experienced staff not only exacerbates workforce shortages but also erodes institutional knowledge and continuity of care [18]. These findings underscore the urgent need for healthcare systems to invest in long‐term well‐being strategies, including mental health support, leadership development, and structural reforms that prioritize staff safety and sustainability, especially in times of crisis. These are lessons we must carry forward post pandemic.

In a recent Australian Government Covid‐19 Parliamentary enquiry, there is recognition that HCP came under considerable strain during the pandemic [19]. Indeed, HCPs during pandemic conditions are most at risk of the acquisition of the virus at rates higher than the community [20], increased stress and burnout [21], anxiety and depression [7], and leaving the profession altogether [17]. The rapid nature of communication, directives and protocols, left Australian HCPs feeling vulnerable, unsure, and in crisis. While the community at large felt the effects of COVID‐19 restrictions, it could be argued that HCPs are among the most impacted in the Australian society [19]. This impact posed a significant challenge for Australian healthcare, which was already under immense pressure and workforce shortages. This study highlights that the care and support for frontline HCPs during and post the pandemic is an essential area of inquiry and should be of greatest import to healthcare providers and governments. While the health and well‐being of HCP has been recognized as essential for future pandemic planning and preparedness, more resourcing needs to be allocated to supporting the mental and physical well‐being of the health workforce [19].

A specific aspect that impacted the mental and physical well‐being of HCPs in this study was burnout. Burnout of HCPs is well documented as an outcome from the COVID‐19 pandemic [5, 22]. This study provides valuable context to larger studies, such as that of Smallwood et al. [6], and provides insights into the reasons for such burnout. Given the gravity of the impacts of pandemics, prevention of ways to reduce burnout is worthy of future exploration and consideration. Indeed, the lessons from this study may provide insights into leaders and policymakers to ensure that future initiatives and processes meet the needs of those at the frontline.

The uncertainty of the pandemic, and the fact that Australian healthcare was largely underprepared, necessitated the need for innovation and opened a space to do so like never before. The silver lining for healthcare administrators is that the lessons learned from COVID‐19 provide abundant avenues for system redesign, innovation, and negotiation. Necessity is often the trigger for innovation, and this was seen across the Australian healthcare system, in reformed models of care, resource building, and operational/funding changes to meet local needs [3]. The participants in this study provided examples of thinking outside the box, innovating, and bending traditional structures to navigate the challenges. While some innovation and changes to practice and models of care were positive, in some instances, the frequency of changes and reduction of nonfrontline HCPs caused concerns due to the reduced consultation with the HCPs these changes would affect [23].

The themes identified in this study resonate strongly with recent qualitative research exploring healthcare worker experiences during the COVID‐19 pandemic. Chemali et al. [24] found that HCW experiences were shaped by multilevel factors—individual, institutional, and policy—which aligns with our participants’ accounts of fractured leadership, personal vulnerability, and inconsistent messaging. Similarly, Page et al. [25] reported that Australian health professionals often rely on emergent, ad hoc strategies to cope with system pressures, mirroring the innovative workarounds and peer support described in our themes. Zhang and Gu [16] highlighted the inadequacy of long‐term psychological support for HCWs, a concern echoed in our participants’ reflections on burnout, trauma, and the lack of meaningful postcrisis care. These parallels reinforce the validity of our findings and underscore the urgent need for structural reform and sustained investment in workforce well‐being.

This study contributes to the growing body of international literature, which captures the lessons learned from HCPs’ experiences during the COVID‐19 pandemic. Orhierhor et al. [26] highlighted the importance of bidirectional communication between policymakers and frontline workers, noting that inconsistent messaging and lack of involvement in planning undermined policy implementation. Our study strengthens this need, further evidencing the need for clear and regular communication channels. Similarly, Busis et al. [27] emphasized that the pandemic exacerbated pre‐existing burnout and moral injury among healthcare workers, calling for systemic reforms to prioritize clinician well‐being and resist a return to “business as usual” (para. 5). The study has demonstrated how clinicians can be innovative, reflexive, and responsive to rapid changes, factors that should be considered in future planning.

Furthermore, analyses of healthcare resilience frameworks have also revealed the need for multilevel, interdisciplinary approaches to preparedness, moving beyond physical capacity to include relational and adaptive dimensions of resilience [28]. Indeed, Ellis et al. [29] identified eight key capacities, such as leadership, coordination, and learning, that not only supported resilient performance but also noted the emotional toll on healthcare workers who bore the burden of constant adaptation. Such lessons are important to reflect upon and build into future planning.

In many ways, the lessons learned from the pandemic have accelerated innovation. The World Economic Forum [30] underscored the pandemic’s role in accelerating innovation, particularly in digital health and integrated care models, offering replicable strategies for strengthening health systems in low‐ and middle‐income countries. These studies collectively reinforce the urgency of embedding frontline insights into future planning and of sustaining the innovations and support mechanisms that emerged during the crisis.

Frontline HCPs, in this study, were at the forefront of innovation, illustrating how this cohort of workers are key to identifying and negotiating problems as they see them. Policymakers must take note of these lessons learned and consider how structures can support such agile innovation in the future. Indeed, questions that should be asked now include How can we maintain the space for innovation in healthcare? How do we ensure these findings are captured and built into future planning? How can frontline healthcare workers lead the change in this postpandemic world? Future areas of inquiry might focus on these points.

### 4.1. Limitations

This study recruited nine participants, the minimum small sample size described by Hennick and Kaiser [13] for qualitative research. It is possible that participants were less inclined to participate in 2023 when we were 3 years into the pandemic. There is no doubt that the COVID‐19 pandemic was deeply disruptive to HCPs. A sense of needing to move on and adapt was evident; thus, participants may have not wanted to engage in reflection about this difficult time.

While the overall number of participants may limit the application of findings, this study was important for adding perspectives beyond the initial months of the pandemic. Each participant offered deeply personal and professional reflections, contributing valuable perspectives that enrich our understanding of the healthcare workforce’s challenges during the pandemic. Their willingness to share their experiences, often recounting trauma and transformation, underscores the importance of capturing these voices in postpandemic inquiry.

## 5. Conclusion

This study provides insight into the experiences of Australian HCPs working directly with patients during the COVID‐19 pandemic. The findings suggest that participants experienced feelings of vulnerability and fear as they grew to understand the risks of the pandemic, sentiments that have now been broadly captured within the literature. These experiences hold opportunities for services to reflect, learn, engage, and codesign systems, which are future proof should the system experience similar disruptions. Anxiety and stress in the workplace were reported to be driven by a lack of clear and coordinated messaging within and across health services. Limited resourcing and ineffective leadership further exacerbated the feelings of chaos and burnout, with some participants describing their experiences as traumatic; this has serious implications for future policy, leadership, and for the well‐being of HCPs. Further exploring available supports for HCPs to address ongoing feelings of burnout and trauma is important to reducing further attrition in the workforce. In addition, the lessons learned from the experience of HCPs need to be incorporated into future pandemic planning and preparedness.

## Funding

There was no funding support for this research. Open access publishing was facilitated by the Central Queensland University, as part of the Wiley–Central Queensland University agreement via the Council of Australasian University Librarians.

## Conflicts of Interest

The authors declare no conflicts of interest.

## Supporting Information

Supporting information Supporting File 1: COREQ Checklist—COVID‐19 study.

## Supporting information


**Supporting Information** Additional supporting information can be found online in the Supporting Information section.

## Data Availability

The data that support the findings of this study are available on request from the corresponding author. The data are not publicly available due to privacy or ethical restrictions.
